# Adsorption of Mussel Protein on Polymer Antifouling Membranes: A Molecular Dynamics Study

**DOI:** 10.3390/molecules26185660

**Published:** 2021-09-17

**Authors:** Fengfeng Gao

**Affiliations:** Department of Chemical Engineering, Zibo Vocational Institute, Zibo 255300, China; 11364@zbvc.edu.cn; Tel.: +86-533-2348221

**Keywords:** antifouling membrane, protein adsorption, mussel protein, hydrophilic film, hydrophobic film

## Abstract

Biofouling is one of the most difficult problems in the field of marine engineering. In this work, molecular dynamics simulation was used to study the adsorption process of mussel protein on the surface of two antifouling films—hydrophilic film and hydrophobic film—trying to reveal the mechanism of protein adsorption and the antifouling mechanism of materials at the molecular level. The simulated conclusion is helpful to design and find new antifouling coatings for the experiments in the future.

## 1. Introduction

Marine fouling is one common problem for ships and marine facilities. The adhesion of marine organisms leads to the increase of hull surface roughness, which can increase the resistance of navigation, and the organic acids released by these organisms also accelerates the corrosion of ships and marine facilities. Worldwide, the cost of fuel consumption, hull cleaning, painting, and maintenance caused by marine fouling is about several billions of dollars per year.

In order to reduce the loss caused by marine fouling, the common method is to apply antifouling coating on the surface of ship. The traditional antifouling coating is mixed with organic copper, organic tin, etc., and these coating are mainly to kill the marine organisms attached to the hull surface by releasing heavy metal ions. However, these heavy metal ions accumulate in the food chain and eventually endanger human beings [1]. At present, designing and developing new environmentally friendly marine antifouling coatings has become a hot new trend in the field of biological antifouling.

There are many kinds of adhesion marine organisms in the ocean, including algae, shellfish, sponge, etc. Among them, shellfish (such as barnacles, mussels, etc.) are more difficult to treat. Normally, the attachment mechanism of them depends on their own released proteins temporarily or permanently adhering to the solid surface (such as the hull). At present, the adhesion materials being widely studied are barnacle glue and mussel protein in most of research experiments. In these experiments, the adsorption of proteins on the surface of materials is related to their own properties (such as surface charge, hydrophobicity, conformation, etc. [2,3]) and the materials surface’s properties (such as surface roughness, chemical composition, etc.).

Till now, there has not been a unanimous view about antifouling mechanism. However, it is generally known that the hydration layer on the surface of materials has a great relationship with its antifouling effect [4,5,6]. A large number of experimental studies show that the antifouling effect of a hydrophilic surface is better than that of a hydrophobic surface [7,8]. This contributed that one closely bonded hydration layer can be formed on the surface of hydrophilic materials, and the adsorption process of proteins must destroy the hydration layer firstly. Therefore, the binding ability between water molecules and membrane materials can reflect the difficulty of binding between proteins and solid surfaces [9,10,11,12].

It is very important to understand the interaction mechanism between protein and solid surface for both theoretical and applied research. In order to design and find new antifouling materials, it is necessary to study the adsorption mechanism between biological proteins and antifouling materials. However, it is very difficult to reveal the internal mechanism of adsorption at the molecular level for any experimental technique. In this paper, we used molecular dynamics method to study the adsorption behavior of mussel protein on different materials surface, and we will try to explain the adsorption mechanism of mussel protein and the antifouling mechanism of the material surface on the microscopic level.

## 2. Simulation Method

Polymer antifouling membrane is constructed by Amorphous cell module in Materials Studio Software (version 4.4). Firstly, the dimethylsiloxane (PDMS) layer was constructed. Its density is 0.965 g/cm^3^ and the thickness of the membrane is about 2 nm. Then, alkyl chains of -(CH_2_)_10_CH_3_ and -(CH_2_)_10_COOH) are grafted on the surface of PDMS, respectively, and the grafting number density is about 0.66 per square nanometer. Finally, CH_3_-SAM and COOH-SAM polymer antifouling membrane are obtained. Both represent the hydrophobic and hydrophilic antifouling membrane, respectively [13,14,15,16].

**Figure 1:**
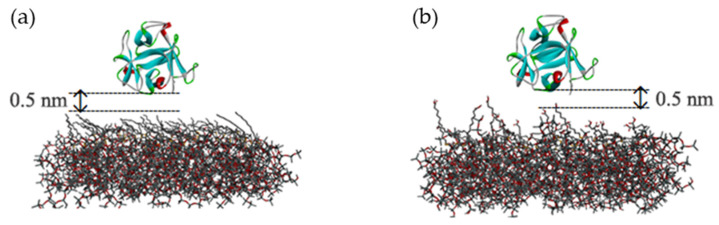
Initial configuration of system: (**a**) protein on the surface of CH3-SAM membrane; (**b**) protein on the surface of COOH-SAM membrane.

In the simulation, mussel protein (PDB ID: 5DUY) is used as the model protein, which contains 150 amino acid molecules. It is similar to the spherical structure and contains a typical secondary structure. At first, mussel protein is placed 0.5 nm above the surface of the two antifouling membranes (shown in Figure 1), and six chloride ions are randomly placed as counterions to balance the positive charge of the protein. The same thickness of water layer is added above the two polymer membranes. In order to eliminate the possible high energy caused by conformational overlap, the water molecules in the range of 0.2 nm around the protein and polymer membranes are deleted [17,18].

In the simulation, the united-atomic force field GROMOS 45a3 is selected [19] and the software package GROMACS (version 4.5.5), is carried out to run the molecular dynamic calculation [20,21]. First of all, for the initial configuration, the steepest descent method was performed several hundred steps to eliminate conformational overlap; then, the NVT ensemble was run for at least 25 ns to obtain the equilibrium of system; then, another 75 ns MD simulation was run to find out the statistical information about mussel protein and the antifouling membrane. During the simulation, in order to reduce the simulation time, the PDMS layers are fixed, and the periodic boundary conditions in XYZ directions are used. For the solvent water, the single point charge (SPC) model is selected [22]. In the simulation, the PME method was used to handle the long-range electrostatic interaction [23], and the radius of non-bond interaction was 1.2 nm. The Berendsen method was used to control the temperature [24], and the LINCS method was selected to constraint the bond of molecule [25]. The simulated step was 2 fs, and the trajectory of system was stored each 100 ps. In the production of simulation, GROMACS analysis program is used to analyze the simulation results, and VMD software (version 1.9.3), is used to visualize the molecular dynamics trajectory.

## 3. Results and Discussions

### 3.1. Adsorption Process

**Figure 2:**
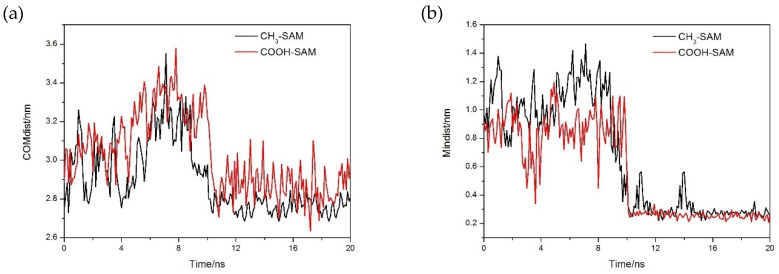
(**a**) The variation of the distance between mussel protein core and antifouling membrane with time; (**b**) the variation of the minimum distance between mussel protein and antifouling membrane with time.

In Figure 2, the centroid distance between mussel protein and polymer membrane was calculated, and the variation of minimum distance with time evolution was shown. It is obvious that the distance between mussel protein and polymer membrane for the two systems decreased rapidly and reached equilibrium at a short simulated time, indicating that mussel protein can reach a stable adsorption state for the present simulated model at a short simulated time. We noted that the distance between protein and membrane fluctuated greatly, which indicated that protein constantly adjusted its own configuration during the process of adsorption until an optimal site for adsorption was finally obtained [13,14,15,16].

**Figure 3:**
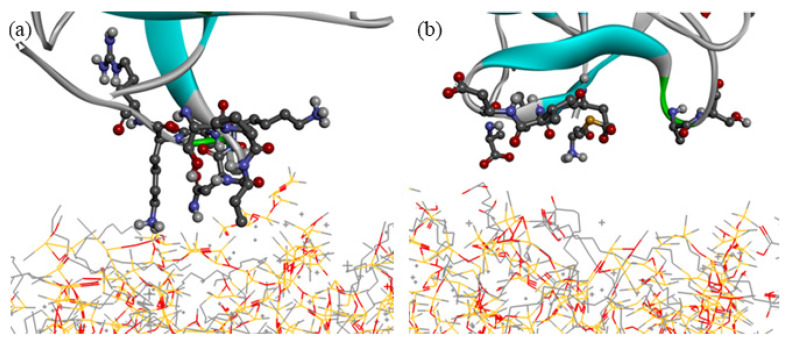
Partial enlarged drawing after stable adsorption. (**a**) CH_3_-SAM surface of self-assembled membrane. (**b**) COOH-SAM membrane surface.

**Figure 4:**
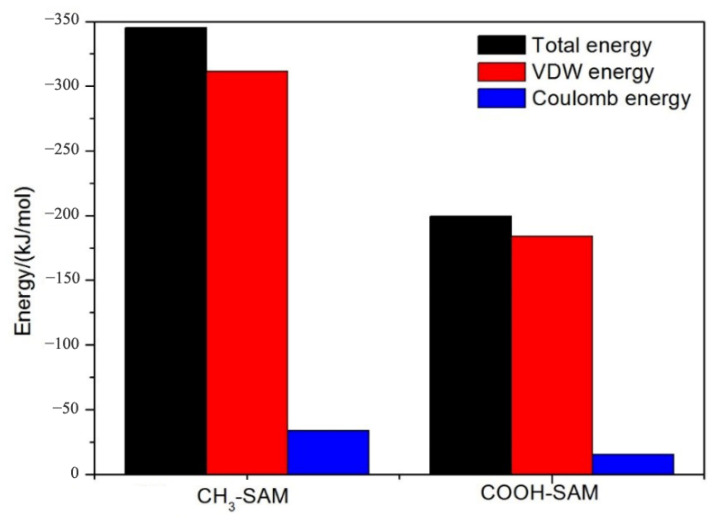
Energy diagram of non-bond interaction between mussel protein and substrate.

After the adsorption equilibrium of protein was obtained, the residue types of amino acid of protein on the CH_3_-SAM and COOH-SAM polymer antifouling membrane can be divided, and the results are shown in Table 1. By comparing the residue types of mussel proteins on different self-assembled membrane surfaces, we noted that the nonpolar residues are major on the surface of CH_3_-SAM membrane, while the polar residues are major on the surface of COOH-SAM membrane (Figure 3). We speculate that the surface of protein contains hydrophilic polar residues, and the hydrophobic nonpolar residues are mostly in its interior of spherical protein. When the protein interacts with the CH_3_-SAM membrane, the hydrophobic residues can be turned over from the interior and form the better combination between the hydrophobic CH_3_-SAM surface and hydrophobic residues of protein. In order to prove the speculation, we calculated the interaction energy between mussel protein and two self-assembly membranes, respectively. As shown in Figure 4, it is obvious that the non-bond interaction energy between CH_3_-SAM membrane and mussel protein is greater than that between COOH-SAM membrane and the protein. This shows that the hydrophobic surface has a stronger effect on mussel protein molecules, and the adsorption of mussel protein on its surface is more stable and difficult to be separated. At the same time, we noted that the proportion of van der Waals (VDW) interaction is much larger than that of Coulomb interaction during the simulation process. For CH_3_-SAM surface, the VDW energy is about 320 kJ/mol, which is approximately 94% contribution to the total energy. While the VDW energy for COOH-SAM surface is about 180 kJ/mol, contributing to 90% to the total energy. These indicate that the driving force of protein adsorption is mainly van der Waals interaction between protein and membrane [26].

### 3.2. Properties of Hydration Layer on Membrane Surface

The antifouling ability of materials is closely related to the surface hydration layer [4,27]. The hydration layer can act as a physical barrier when the protein is close to the surface of material, and we evaluate the antifouling ability of the two-polymer membrane by analyzing the structure and stability of the hydration layer.

**Figure 5:**
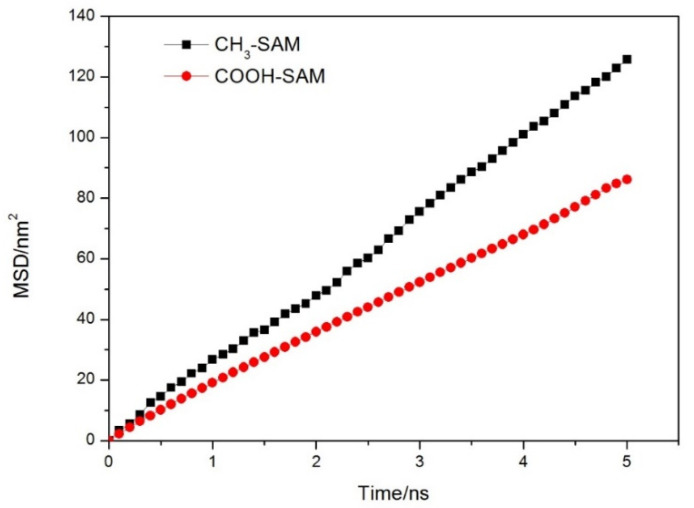
Relation diagram of the variation of average azimuthal shift with time of self-assembled membrane surface hydration layer molecules.

Firstly, the water molecules in the range of 0.4 nm on the surface of membrane were defined as the hydration layer. The mean square displacements (MSD) of water molecules in hydration layer with time evolution for the two investigated systems are shown in Figure 5. By fitting the two curves and calculating their slopes, the diffusion coefficient (D) of water molecules in the hydration layer can be calculated by Equation (1):

(1) $$D=\frac{1}{2dN}\underset{t\to \infty }{\lim}\frac{d}{dt}\overset{N}{\underset{i=1}{\sum }}\langle {\left[\vec{r_{i}\left(t\right)}-\vec{r_{i}\left(0\right)}\right]}^{2}\rangle$$

where *N* represents the number of target molecules in the system, $\vec{r_{i}\left(0\right)}$ and $\vec{r_{i}\left(t\right)}$ represent the coordinates of the ith particle at 0 and *t*, respectively.

Table 2 lists the Ds of water molecules in the hydration layer and bulk phase. It was found that the D of water molecules in the hydration layer decreased compared with that in the bulk solution for the two systems. It indicates that the interaction between the surface of materials and water molecules restricted the diffusion of water molecules on the membrane surfaces, especially for the COOH-SAM system. It also shows that the binding ability of COOH-SAM self-assembly membrane to water molecules is relatively stronger. Meanwhile, we also noted that the D of water molecules in the vertical direction is much lower than that in the horizontal direction, indicating that the solvent layer molecules are difficult to separate from the surface of the self-assembled membrane.

Relaxation time can describe the limiting ability of antifouling membrane to the molecules of hydration layer. The longer the relaxation time is, the stronger the binding ability of antifouling membrane to water molecules is and representing the better antifouling effect. Its value can be obtained by fitting autocorrelation function [28,29,30]:

(2) $$C_{r}\left(t\right)=\frac{1}{N_{w}}\overset{N_{w}}{\underset{j=1}{\sum }}\frac{\langle P_{Rj}\left(0\right)P_{Rj}\left(t\right)\rangle }{{\langle P_{Rj}\left(0\right)\rangle }^{2}}$$

where $P_{Rj}$ represents a binary operator, if the target molecule *j* remains in the initial range at time *t*, then $P_{Rj\left(t\right)}=1$, if not, then $P_{Rj\left(t\right)}=0$. $N_{w}$ is the total number of target molecules in the initial range defined by us, < > represents ensemble average.

**Figure 6:**
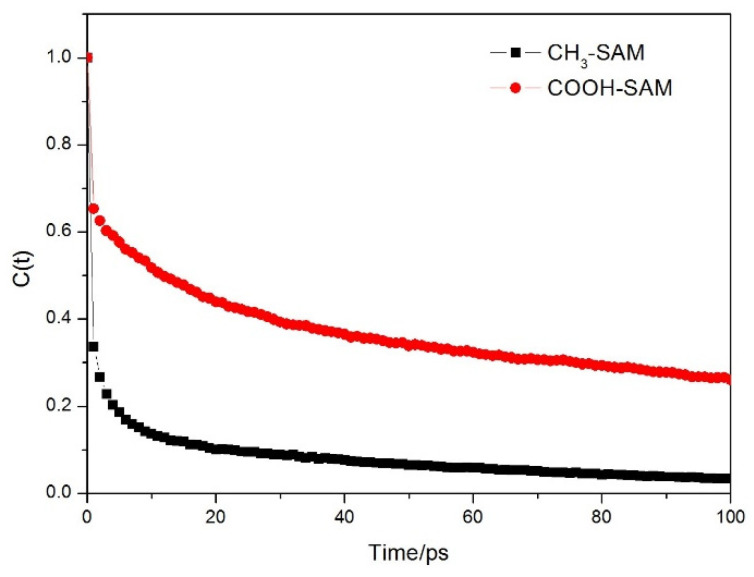
Autocorrelation function of molecules in hydration layer on polymer membrane surface.

Figure 6 shows the relationship of *C(t)* and the time t. It can be seen that the autocorrelation functions of water molecules on the two membrane surfaces show the same trend of decay, and the decay of water molecules on CH_3_-SAM membrane surface is relatively fast. Fitting the curve using the equation $C_{r}\left(t\right)=A_{r}\exp\left(-t/\tau_{\mu }\right)$, the relaxation time *τ_μ_* can be calculated. Table 2 lists the *τ_μ_* of hydrated layer molecules and bulk water for the different self-assembled membranes. Due to the existence of polymer membrane, the relaxation time of the surface hydration layer molecules is longer than that of the bulk phase water. It explains that both polymer membranes have limiting effects on the surface hydration layer molecules. The COOH-SAM membrane has greater limiting effect on the hydration layer molecules. Meanwhile, the number and life of hydrogen bonds (HBs) formed between water molecules and self-assembled membrane are also listed in Table 2. The data showed that the number of HBs formed between water molecules and COOH-SAM polymer is relatively stronger, and the life of HBs is relatively long. These also explained the reason why water molecules and COOH-SAM polymer can form the strong hydrogen bonding structure.

### 3.3. Adsorption Mechanism

**Figure 7:**
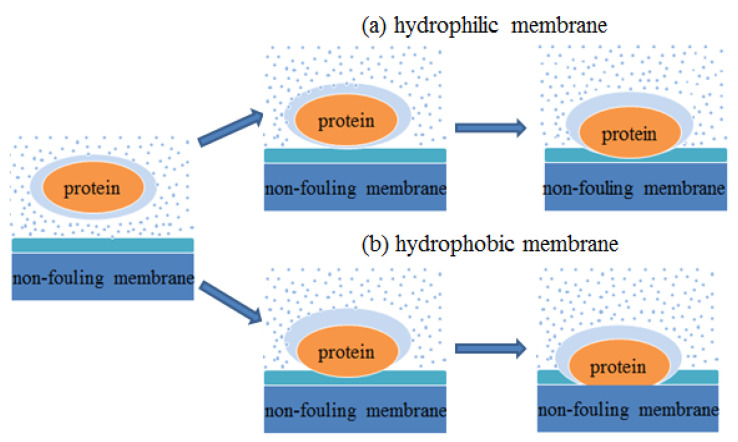
Antifouling mechanism. (**a**) Adsorption process of protein on hydrophilic membrane surface. (**b**) Adsorption process of protein on hydrophobic membrane surface.

In the aqueous environment, a close hydration layer can be formed between mussel protein and antifouling membrane. When the protein molecules in the aqueous approach to the membrane surface, they must destroy the hydration layer first, that is, the adsorption of protein molecules on the antifouling membrane surface is essentially the competitive adsorption behavior between protein molecules and water molecules on the interface. As shown in Figure 7, during the adsorption process, the mussel protein first exposes the hydrophobic residues to the surface through its own structure changes. In this process, the exposure of hydrophobic residues damaged the hydration layer on the surface of the protein. When the protein touches the hydration layer on the membrane surface, in order to complete the adsorption, the energy barrier brought by the hydration layer of the antifouling membrane must be overcome. Due to the different hydrophilicity of antifouling membrane surface, the structure and properties of the surface hydration layer are different. For the hydrophilic carboxyl self-assembled membrane, because the surface contains hydrophilic functional groups, the interaction with water molecules is stronger, and the formed hydration layer is also tighter, so the energy barrier that they should overcome in the process of mussel protein adsorption is larger, which is not conducive to the combination of protein molecules and membrane. However, for the hydrophobic methyl self-assembled membrane, the interaction between the surface and water molecules is weaker, and the formed hydration layer is relatively loose. The mussel protein can be adsorbed on the surface of the antifouling membrane by overcoming the smaller energy barrier, and they form a more stable combination through the hydrophobic interaction.

## 4. Conclusions

The adsorption behavior of mussel protein on the surfaces of two antifouling materials was studied by molecular dynamics simulation. By analyzing the adsorption process, including the distance between the protein centroid and the membrane, the type of residues near the adsorption site, the interaction energy between the protein and the antifouling membrane, the diffusion properties of the hydration layer molecules on the membrane surface and the life of HBs, the following simulated conclusions are listed:

(1) In the process of protein adsorption on the surface of different materials, influenced by the chemical composition and structure of the material surface, it will deform through the rotation of its own skeleton, so as to separate the hydration layer on the surface from the protein and form a stable binding with the material surface at the optimal site.

(2) The interaction between mussel protein and antifouling membrane is mainly van der Waals interaction, and the binding between mussel protein and methyl self-assembled membrane is relatively stable.

(3) When mussel protein is adsorbed on the surface of carboxyl self-assembled membrane, it needs to overcome the energy barrier brought by the dense hydrated layer polarized on the surface of the membrane. Compared with the methyl self-assembled membrane, it has better antifouling performance.

In conclusion, this paper uses molecular dynamics method to compare and study the adsorption process of mussel protein on the surface of COOH-SAM membrane and CH_3_-SAM membrane and reveals the factors that hydrophilic self-assembled antifouling membrane has better antifouling characteristics from the molecular level, which is of great significance for optimizing and designing new antifouling coatings.

## Figures and Tables

**Table 1:** Statistics of residue types of mussel protein on polymer membrane surface.

System	Polar (%)	Nonpolar (%)
CH_3_-SAM	21.05 ± 0.05	78.95 ± 0.05
COOH-SAM	71.42 ± 0.05	28.75 ± 0.05

**Table 2:** Some property parameters of solvent layer molecules on the substrate surface.

System	Diffusion Coefficients (Ds) × 10^−5^ (cm^2^ s^−1^)			HBs Life (ps)	HBs Num (nm^2^)	τ_μ_ (ps)
	D	D_⊥_	D_//_			
CH_3_-SAM	3.21 ± 0.20	0.24 ± 0.43	3.99 ± 0.01	74.34	0.13	46.78
COOH-SAM	2.56 ± 0.09	0.19 ± 0.43	3.73 ± 0.13	129.88	0.25	68.82
Bulk water	3.66 ± 0.04	3.58 ± 0.19	3.69 ± 0.04	-	-	23.93

## Data Availability

The data presented in this study is available upon reasonable request.
